# Mechanism insight into the conversion between COS and thiophene during CO_2_ gasification of carbon-based fuels

**DOI:** 10.1038/s41598-024-67180-w

**Published:** 2024-07-10

**Authors:** Shengxian Xian, Ye Xie, Qing Xu, Zhisi Yang, Haowei Li, Yujian Wu

**Affiliations:** 1 College of Ocean Engineering and Energy, Guangdong Ocean University, Zhanjiang, 524088 China; 2 Guangdong Provincial Key Laboratory of Intelligent Equipment for South China, Sea Marine Ranching, Guangdong Ocean University, Zhanjiang, 524088 China

**Keywords:** Carbon-based fuel, CO_2_ gasification, Sulfur transformation, Conversion between COS and thiophene, Fossil fuels, Renewable energy

## Abstract

Thiophene is the organic sulfur with good thermal stability in carbon-based fuel, clarifying the conversion mechanism between thiophene and COS is beneficial for achieving in-situ sulfur fixation during CO_2_ gasification of carbon-based fuels, but the mechanism has rarely been reported. Therefore, calculations based on density functional theory were performed and 16 reaction paths were proposed in this research, clarifying the decomposition mechanism of thiophene and re-fixation mechanism of COS. The attachment of CO_2_ will lead to the destruction of the thiophene ring and the generation of COS, and CO_2_ adsorption is the rate-determined step, while the carbon atom that adjacent sulfur atom is the reaction active site. However, the energy barriers of CO_2_ addition reactions are lower than those of CO_2_ adsorption reactions, and the energy barrier of reactions occurring on the aliphatics are lower than that occurring on the aromatics. The combination of CO_2_ and thiophene will thermodynamically lead to the generation of COS and CO. Moreover, gaseous sulfur generated from thiophene decomposition will be converted mutually, while H_2_S will not be converted into COS. Furthermore, COS will be captured by char, forming solid organic sulfur. The re-fixation of COS will occur on aliphatic chains from the decomposition of aromatics.

## Introduction

Coal is the important pillar for current world economic development, while bio-energy is one of the promising net zero carbon energy^1,2^. CO_2_ gasification of the above solid carbon-based fuel for producing gaseous fuel, is conductive for CCUS (Carbon Capture, Utilization and Storage) and fuel quality improvement. However, sulfur in carbon-based fuel will be released during CO_2_ gasification, forming gaseous sulfur and further reducing the quality of coal gas/bio-gas. Calcium-based desulfurizer is beneficial for the source fixation of sulfur, but it will lead to the low-temperature melting of ash and secondary pollution of char during fluidized-bed gasification^3^. Besides calcium-based desulfurizer, gaseous sulfur captured by nascent char were also been observed^4^, but the underlying mechanism has not yet been reported. Thiophene is one of the organic sulfur with good thermal stability, and it can be regarded as the final product of sulfur fixation by char^5^. Clarifying the conversion mechanism between thiophene and gaseous sulfur is beneficial for achieving in-situ sulfur fixation.

Density functional theory (DFT) has been widely used to reveal the reaction mechanisms at the molecular level, and the sulfur transformation during the thermal conversion of carbon-based fuels have been studied. Yang^6^ investigated the conversion mechanism of thiophene into H_2_S, and claimed that the decomposition of thiophene tended to be triggered by hydrogen transfer between adjacent carbon atoms, and further hydrogen transfer and breakage of C–S bond led to the generation of H_2_S. Liu^7^ discussed the effect of steam on the thiophene pyrolysis, and reported the conversion to H_2_S was promoted by steam, because the addition of steam catalyzed hydrogen transfer and saturated the thiophene ring. Similarly, Lu^8^ studied the desulfurization mechanism of thiophene compounds in supercritical water, and found out that the introduction of H_2_O promoted the breakage of C-S bond, and thus promoting the generation of H_2_S. Zhang^9^ compared the decomposition pathways of 2-methylthiophene during inert and oxidizing atmospheres, and reported that the desulfurization of 2-methylthiophene is thermodynamically more favorable in oxidizing atmosphere. Valuable conclusions have been obtained in published researches, H_2_ evolution and its relationship with the released sulfur have also been studied^10–13^, while the re-fixation of H_2_S has also been investigated in previous research^14^,the conversion mechanism of thiophene into H_2_S in different atmospheres have been elucidated. However, the decomposition mechanism of thiophene into COS have rarely been reported, while COS is the dominate gaseous sulfur during CO_2_ gasification of carbon-based fuels^15^, and the conversion mechanism of COS to thiophene in CO_2_ atmosphere is not yet clear. Therefore, revealing the conversion mechanism between thiophene and COS is necessary, clarifying the sulfur migration pathway in CO_2_ gasification is conducive for laying the foundation for in-situ sulfur fixation.

The conversion mechanism between thiophene and COS were studied using DFT (density functional theory) calculations of conversion between thiophene and COS during CO_2_ pyrolysis were analyzed in ORCA program with function M06-2X and basis set def2-SVP/def2-TZVP. The properties of compounds involved in this research were evaluated through the calculations of Mayer bond order (MBO), electrostatic potential and reduced density gradient (RDG), determining the active sites of reactants and revealing the reaction pathways. Furthermore, the occurrence difficulty of reaction was comprehensively evaluated by thermodynamic analysis. The aim of this study is to provide guidance for the source control of sulfur during the CO_2_ gasification of carbon-based fuels.

## Calculation methods

### DFT calculation

All DFT calculations involved in this research were completed using ORCA program^16^.The geometry optimizations of reactants, transition states, intermediates and products were carried out by using the function M06-2X^17^ together with the basis set def2-SVP^18^, and frequency computations were simultaneously carried out to verify the accuracy of each configuration. Furthermore, intrinsic reaction coordinate (IRC) calculation was performed to verify whether a transition structure connects the designated reactants and products. After confirming the correctness of geometry optimization, the electronic energies of materials were calculated at M06-2X/def2-TZVP level, and the Gibbs free energies were further calculated using Shermo program^19^. Gibbs free energies of materials were calculated at 1173K, which is generally the temperature of CO_2_ gasification. The reaction energy can be obtained by the difference of Gibbs free energies between product and reactant, while the energy barrier represents the difference of Gibbs free energies between transition state and reactant.

### Molecular properties analysis methods

Reduced density gradient (RDG)^20^ is beneficial for analyzing the interaction between reactants, and the distribution of electrostatic potential is conductive for predicting active sites on reactants. Mayer bond order (MBO)^21^has been widely used to characterize the relative strength of bonds with same types. The above analyses were performed using Multiwfn and VMD program^22,23^.

## Results and discussion

### CO_2_ adsorption reaction

**Figure 1:**
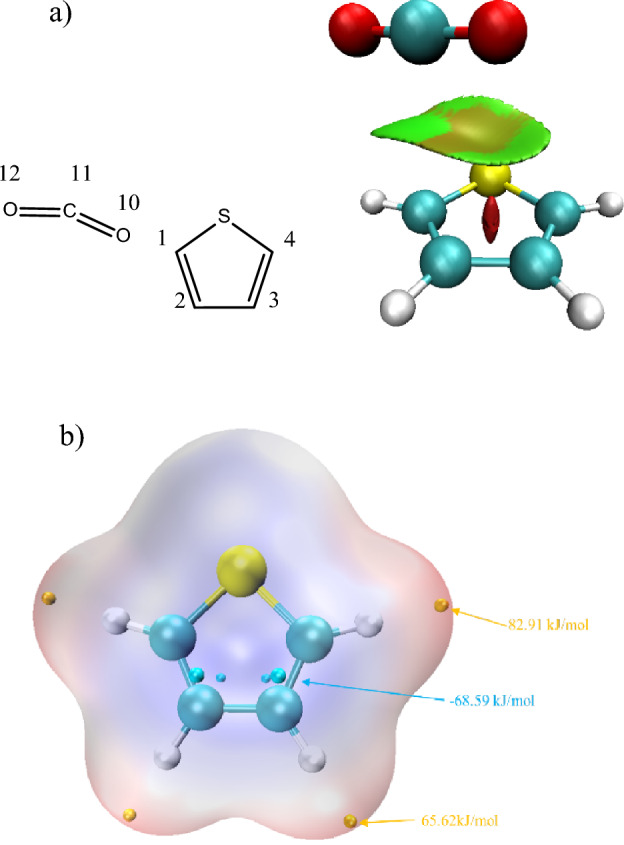
Analysis of reaction system.

**Figure 2:**
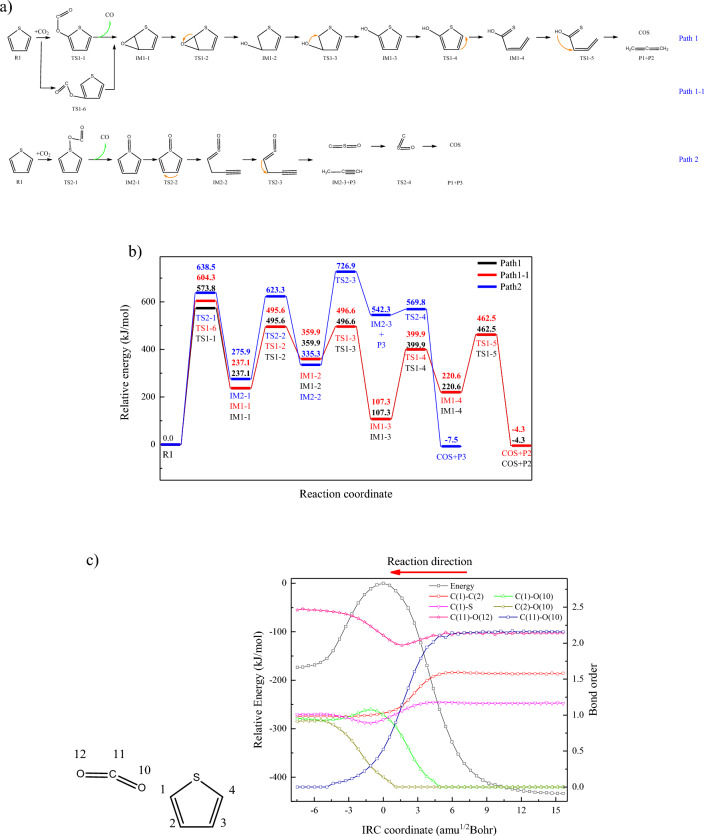
The reaction of CO_2_ adsorption on thiophene. (**a**) The adsorption/desorption reactions of CO2 occur at thiophene ring (**b**) Changes of relative energies in path1-2 (**c**) MBO changes along with the IRC coordinate (CO_2_ adoption on C(1) atom).

CO_2_ adsorption and the formation of CO are the main reactions in the process of CO_2_ gasification, which will lead to the damage of benzene ring^24^. Similarly, the adsorption of CO_2_ on the thiophene ring will also lead to the rupture of the thiophene ring and the formation of COS. RDG analysis of thiophene-CO_2_ system was shown in Fig. 1a. The interaction between thiophene and CO_2_ in the center of thiophene ring is mainly repulsive force, while the interaction between C/S atom on thiophene ring and CO_2_ is mainly attractive force. Thus, the adsorption reactions of CO_2_ occur at C/S atoms are investigated. Reaction paths were shown in the Fig. 2a, and the changes of relative energies were shown in the Fig. 2b. In path 1, the bond angle of CO_2_ (O–C–O) decreases as it approaches thiophene, and one of the C-O bonds was elongated, which is similar to the reactions between CO_2_ and benzene ring^25^. The MBO of C(1)–O(10) increase synchronously as that of C(11)–O(10) decreases, as shown in Fig. 2c. Furthermore, the MBO of C(2)–O(10) begins increasing as that of C(11)–O(10) decreases to 1, and it indicates the rupture of C(11)–O(10) bond enhance the interaction between C(2) and O (10), promoting the formation of ether bond. Meanwhile, the MBOs of C(1)–C(2) and C(1)–S also decrease as the reaction proceeds, destroying the conjugated structure of thiophene ring, generating unstable intermediate with high energy, which will further be decomposed. Ether bond will be further converted to hydroxyl, through the hydrogen transfer between C(1) and O atoms. The hydroxyl will be transfer to C(1) atom, and thus reforming stable thiophene ring, and both of the reaction energy barrier and the relative energy of generated intermediate (IM1-3) are the lowest in Path1. Based on the pyrolysis mechanism of thiophene^6^, the ring-opening reaction can be caused by the hydrogen transfer between C(3) and C(4). Furthermore, COS will be generated through the hydrogen transfer and the rupture of C(1)–C(2) bond, which is similar to the decarboxylation reaction. Moreover, the adsorption of CO_2_ and the desorption of CO will also happen on C(2) atom, generating the intermediate IM1-1 with ether group. The overall reaction path1-1 is basically the same as path1. Besides the CO_2_ adsorption on carbon atoms, the CO_2_ adsorption on sulfur atom was also investigated. Similarly, oxygen atom will be linked to the thiophene ring, through the CO desorption, leading to the generation of sulfinyl group instead of ether group. Subsequently, the ring opening reaction is realized through hydrogen transfer between C(2) and C(3), while strengthening the C(1)–S bond and weakening the C(1)–C(2) bond. Furthermore, the C(1)–C(2) bond will be broke by the hydrogen transfer between C(1) and C(2) atom, forming propargyl and CSO, and CSO will further be transferred into COS. Energy barrier of transformation reaction is 24.5kJ/mol, indicating CSO is unstable and will easily be convert to COS.

According to Fig. 2b, CO_2_ adsorption is the reaction with the highest energy barrier in above three reaction paths, indicating CO_2_ adsorption is the rate-determining step. Comparing the CO_2_ adsorption energy barriers in three reaction sites, the reaction occurs at C(1) atom is the lowest, while that of S atom is the highest. The bond energy of S–O bond and ether bond were calculated, which are 301.12kJ/mol and 479.57 kJ/mol, respectively. It can be inferred that the formation of intermediate IM1-1 is easier than that of IM2-1. Moreover, the electrostatic potential distribution of thiophene, as shown in Fig. 1b, indicates the points with highest electrostatic potential are closed to four carbon atoms rather than sulfur atom, and the point closed to C(1) atom is larger than that of C(2) atom. Thus, the oxygen atom in CO_2_ tends to be adsorbed in C(1) atoms. Furthermore, the ring-opening of thiophene ring is the reaction with the highest energy barrier in the subsequent reactions of path1 and path1-1, which is 292.7 kJ/mol. However, the highest energy barrier in the subsequent reactions of path2 is 391.6 kJ/mol. It can be inferred that the generation of COS from IM1-1 is easier than that of IM2-1, which confirms the above inference that COS generation, causing by CO_2_ adsorption on thiophene ring, follows the rection path1.

CO_2_ adsorption and CO desorption will destroy the aromatic ring, promoting the decomposition of thiophene. However, according to the results above, the energy barrier of CO_2_ adsorption on the thiophene ring is high. Therefore, the CO_2_ adsorption on the non-aromatic structure should be investigated. Hydrogen transfer of thiophene will lead to the destruction of aromatic structure^7^, increasing reaction activity. The hydrogen transfer paths and the corresponding CO_2_ adsorption mechanism are complex^26^, but the formation path of COS is the focus of this research. Thus, the hydrogen transfer reactions happened on C(1) atom are investigated, because C(1) atom(or C(4) atom) is the active site for the CO_2_ adsorption. Hydrogen transfer reactions between adjacent carbon/sulfur atoms are the dominate reactions^6^, and thus the hydrogen transfer reactions between C(1) and C(2) atom ,C(1) and S atom are investigated.

**Figure 3:**
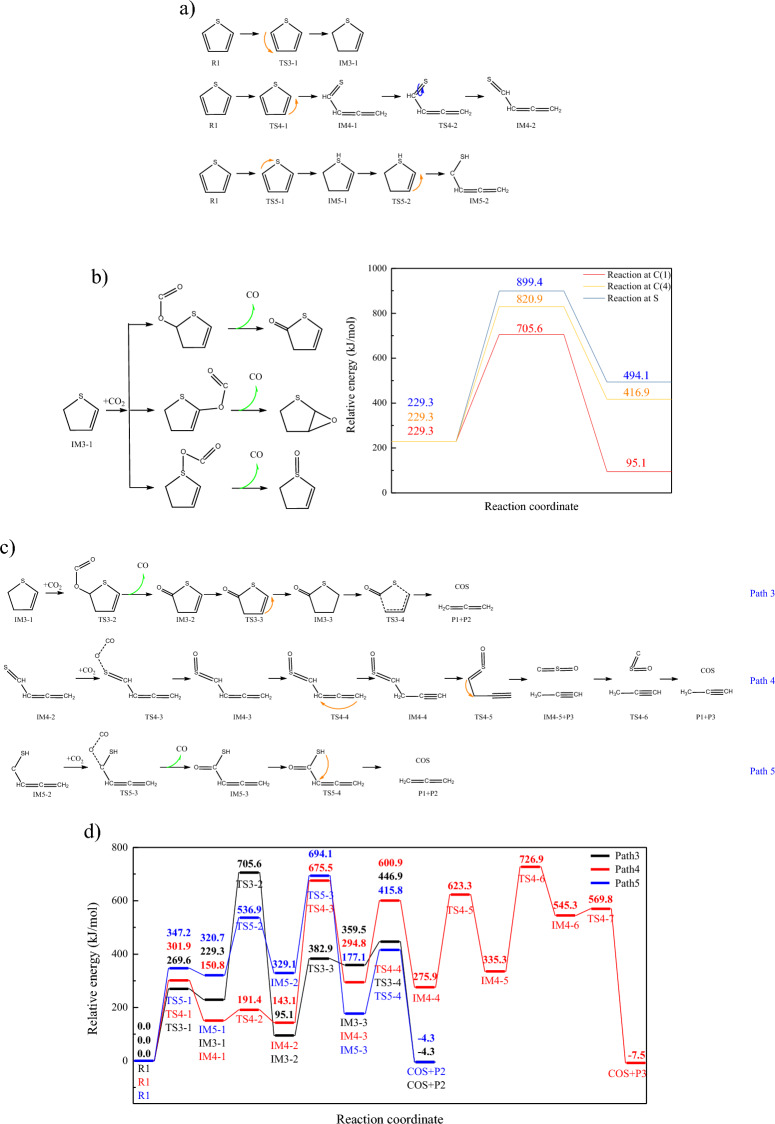
The reaction path3-5. (**a**) Hydrogen transfer reaction paths of thiophene (**b**) CO_2_ adsorption reaction of IM3-1 (**c**) Subsequent reaction of IM3-1,IM4-2 and IM5-2 (**d**) Changes of relative energies in path3-5.

Hydrogen transfer form C(1) to C(2) will not lead to the ring cracking, as shown in Fig. 3a. For IM3-1, CO_2_ adsorption will occur at C(1),C(3),C(4) and S atom, while the reactions occur at C(3) and C(4) are similar to the reaction path1/1–1, and the energy barrier of the reaction occur at C(4) atom is lower. The CO_2_ adsorption path on C(1),C(4) and S atom were shown in Fig. 3b. Energy barrier of the reaction on S atom is the highest, while that of C(1) is the lowest. The sequencing of bond energy is: C=O (980.45 kJ/mol) > ether bond(479.92 kJ/mol) > S=O(354.11 kJ/mol), which is contrary to that of reaction energy barrier, indicating bonding difficulty is the dominate influencing factor of CO_2_ adsorption reaction. Moreover, the MBO of C(1)–C(2) is reduced due to the hydrogen transfer, and the bonding electron of C(1) is converted to lone pair of electron, which is easier to combine with oxygen atom in CO_2_, promoting the formation of C(1)–O bond. Therefore, CO_2_ prefers to be adsorbed at C(1) atom, and the subsequent reaction were shown in Fig. 3c. Hydrogen transfer form C(3) to C(4) atom will weaken C(1)–C(2) and C(4)–S bond, and their MBOs are reduced from 0.91 and 1.02 to 0.84 and 0.93, respectively. The intermediate IM3-3 will be further decomposed into allene and COS.

Hydrogen transfer from C(2) to C(1) atom will lead to the opening of ring, and the subsequent twist of C=S bond generates the active intermediate, as shown in Fig. 3a. The MBO of C(1)–C(2) and C(1)–S in IM4-2 are 1.11 and 1.83, respectively. It indicates the C(1)–C(2) bond is sigma bond, which is stable and difficult to break. Moreover, all electron of C(1) atom in IM4-2 are bonding. Thus, C(1) atom is not the active site for CO_2_ adsorption. Moreover, CO_2_ adsorption reactions on other carbon atoms are not conductive for COS formation. Therefore, CO_2_ adsorption on sulfur atom is investigated, as shown in path 4. Hydrogen transfer from C(4) to C(2) atom in IM4-3 weakens C(1)–C(2) bond, which will be broke through the hydrogen transfer from C(1) to C(2) atom, leading to the decomposition of thiophene and the formation of COS.

Hydrogen transfer from C(1) to S atom will also lead to the opening of ring, as shown in Fig. 3a. Similar to path3, the lone pair of electron of C(1) atom promotes the formation of C=O bond, and the bonding energy of C=O bond is higher than that of S=O. Thus, C(1) is the active site of CO_2_ adsorption in IM5-2, and the subsequent reaction path was shown in path5. The mechanism of subsequent reaction is similar to decarboxylation reaction, IM5-3 will be decomposed to generate COS, through the hydrogen transfer reaction from S to C(2) atom, accompanied with the breakage of C(1)–C(2) bond.

According to Figs. 2b and 3d, the energy barriers of CO_2_ adsorption reactions on S atom are higher than that of C atom. Moreover, the energy barrier of C=O bond formation reactions are 476.2 kJ/mol (TS3-2) and 365.1 kJ/mol (TS5-3), respectively, and they are both lower than the forming energy barrier of ether bond (TS1-1, 573.8 kJ/mol), indicating the non-aromatic structures formed by thiophene pyrolysis are more conducive for CO_2_ adsorption. However, the maximum relative energy in path3 and path5 are higher than that of path1, which may due to the high-energy intermediates generated through thiophene pyrolysis. It can be inferred that reaction rate of path1 is the highest among path1-6. Thus, path1 is the most possible way for COS formation through the CO_2_ adsorption.

### CO_2_ addition reaction

**Figure 4:**
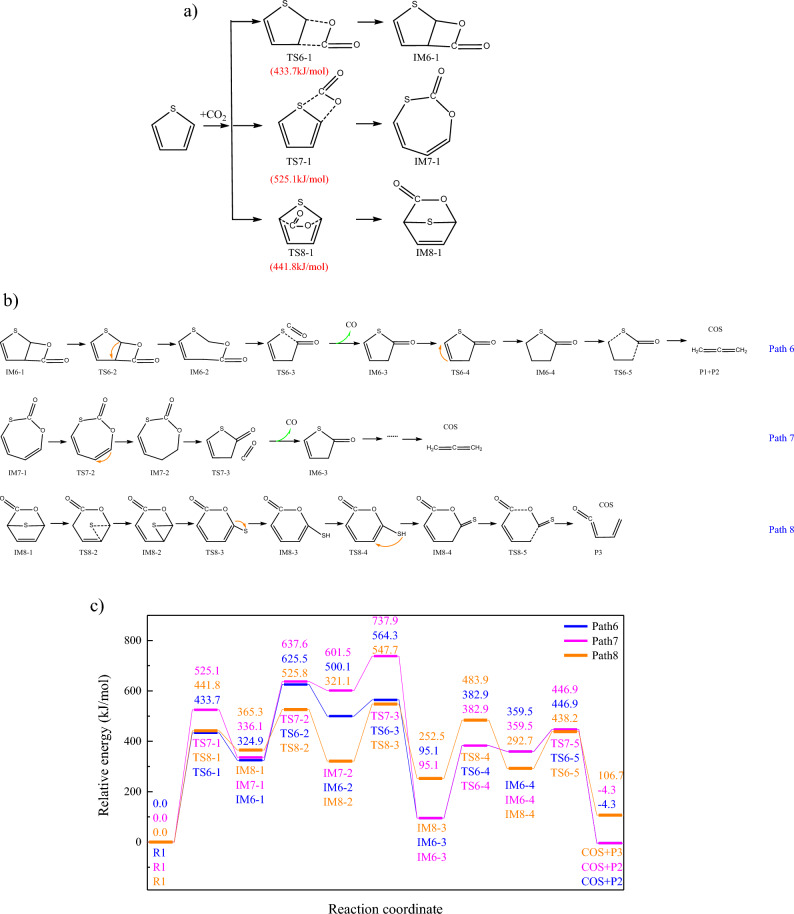
Addition reaction of thiophene and CO_2_. (**a**) CO_2_ addition occurred on thiophene ring (**b**) Subsequent reaction of IM6-1,IM7-1 and IM8-1 (**c**) Changes of relative energies in path 6-8.

Besides adsorption, CO_2_ can also be combined with thiophene ring through addition reaction. Reactions occurring near the S atom is conducive to the rupture of thiophene ring and the formation of COS, and thus the reactions occurring at C(1)–C(2)/C(1)–C(4)/C(1)–C(S) bonds were investigated. Moreover, binding sites of carbon atom in CO_2_ will also influence the reaction pathways, and COS generation pathways were shown in Fig. 4a,b. Energy barriers for the addition reactions are lower than those of the CO_2_ adsorption reactions, and CO_2_ tends to be combined with C(1)–C(2) bonds. The combination of C=C and C=O bonds lead to the generation of bicyclic structure, with poor thermal stability. Bicyclic structure will further be converted to monocyclic structure through the hydrogen transfer. Furthermore, monocyclic organics will be cracked to form CO and five-membered ring, which will be decomposed to form COS. Similarly, the combination of C=C and C=O bonds will also lead to the generation of monocyclic structure, which will further be decomposed to form CO and COS. Moreover, the para-position addition reaction leads to the generation of bridge bond, and the monocyclic structure will be converted to monocyclic structure, forming sulfydryl functional group. COS will be further released through the hydrogen transfer and the breakage of C–C/C-O bonds. Energy changes of path6-8 were shown in Fig. 4c, and the energy barrier of path7 is the highest, while that of path8 is the lowest. However, overall Gibbs free energy change of path8 is positive, while those of path6-7 are negative, indicating the conversion to CO and COS is thermodynamically feasible. The combination of CO_2_ and thiophene ring is the rate determining step for path6-7.

**Figure 5:**
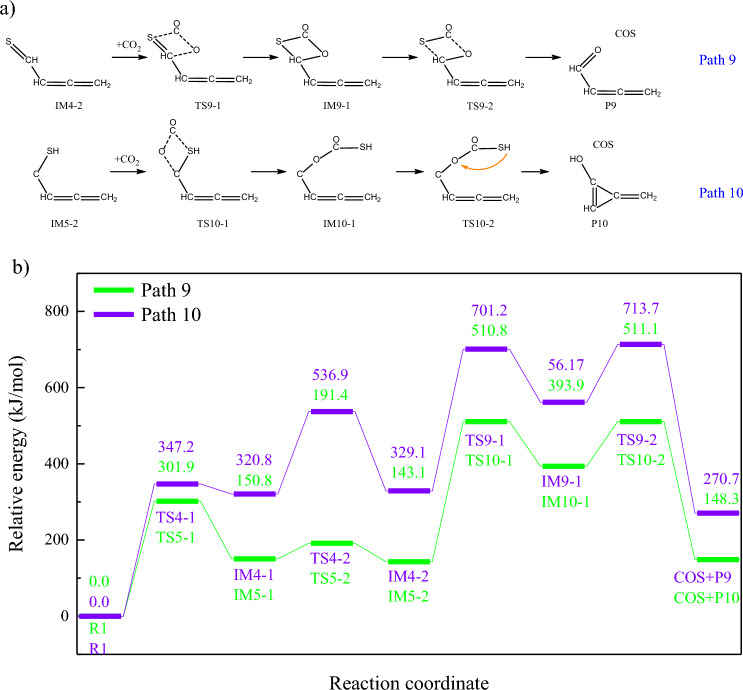
COS formation through the addition reaction of aliphatics and CO_2_. (**a**) Reaction pathways (**b**) Changes of relative energies in path9-10.

Moreover, unsaturated bonds generated from the ring-opening of thiophene will also be combined with CO_2_, through addition reaction, as shown in Fig. 5a. The combination of C=O and C=C bonds will form the quaternary ring, which will be cracked to form COS. Meanwhile, CO_2_ will also be attached to carbanion generated from the ring-opening of thiophene. The combination of C=O and C-S bonds will not form quaternary ring, but promote the breakage of C-S bond. Similar to decarboxylation reaction, further hydrogen transfer leads to the release of COS. Furthermore, according to Fig. 5b, addition reactions are the rate determining steps for path9-10, and CO_2_ is easier to be captured by C=S bond, rather than C-S bond. Moreover, according to Fig. 4b, energy barriers of CO_2_ adsorption reactions are higher than those of CO_2_ addition reactions.

Therefore, during CO_2_ gasification, CO_2_ adsorption will lead to the generation of COS and CO, which is thermodynamically feasible at 1173K. The inference is consistent with the published experimental results^27^, which concluded that the generation of COS is related to the release of CO during the CO_2_ gasification of thiophene. But CO_2_ tends to be combined with thiophene through addition reaction, and the energy barriers of reactions occurring on the aliphatics are lower than that occurring on the aromatics.

### Conversion of gaseous products

**Figure 6:**
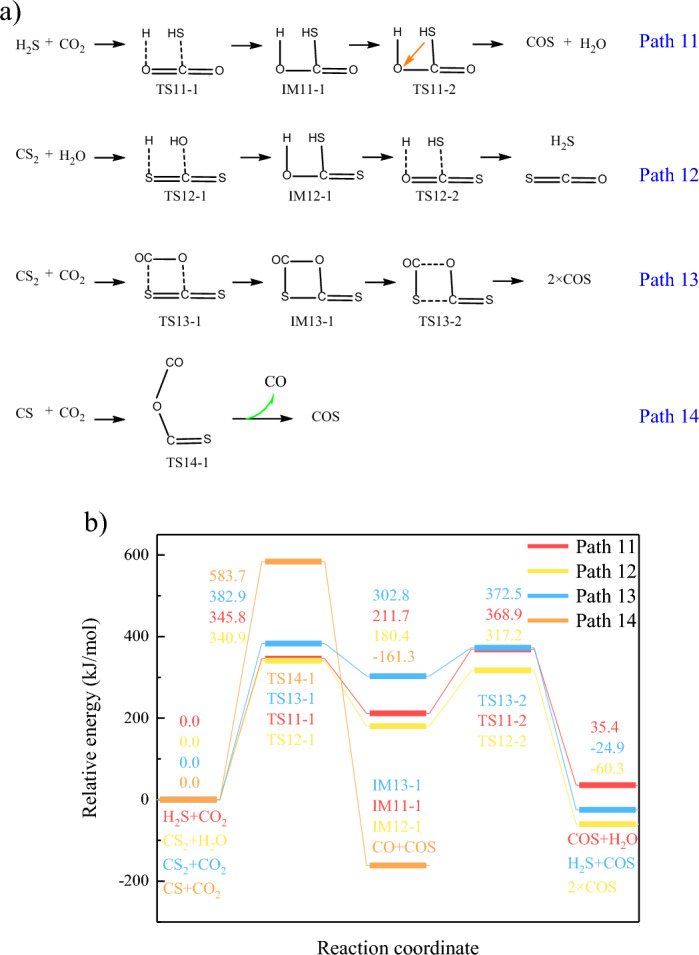
Conversion of gaseous sulfur to COS. (**a**) reaction pathways (**b**) Changes of relative energies in path11-14.

The decomposition of thiophene will generates various gaseous products^6,14^, which will also be converted into COS. The secondary conversion reactions of gaseous products also need to be investigated. During CO_2_ gasification of carbon-based fuels, H_2_S, COS, and CS_2_ are the dominate gaseous sulfur^28^, and thus the conversion of gaseous products (H_2_S, CS_2_, CS) to COS was investigated, as shown in Fig. 6a. H_2_S will be captured by C=O bond in CO_2_ through addition reaction, and further be converted to COS through dehydration reaction. Similarly, H_2_O will be captured by C=S bond in CS_2_, and the generated intermediate will be converted to COS and H_2_S. Moreover, CS_2_ will also be combined with CO_2_ to form quaternary ring, which will further be broke to form COS. Besides addition reactions, CO_2_ will also be attached on CS radical, forming COS and CO through the oxygen transfer.

Furthermore, according to Fig. 6b, COS will be converted into H_2_S during CO_2_ gasification, and the addition of H_2_O is the rate-determining step. The inference is consistent with the published experimental results^29^, which concluded that the key to the catalytic conversion of COS to H_2_S lies in the transfer of H from H_2_O to COS, indicating the addition reaction of H_2_O is the rate determining step. The conversion of CS_2_ and CS to COS are thermodynamically feasible. However, the energy barrier of path14 is the highest, indicating the combination of CS and CO_2_ is difficult. Therefore, during CO_2_ gasification, COS will be converted into H_2_S, while CS_2_ will be converted into H_2_S and COS, with the combination with CO_2_/H_2_O. Moreover, the energy barriers for the conversion of CS_2_ are low, indicating generated CS_2_ will be quickly converted to COS, and thus the sequencing of gaseous sulfur is as follows: H_2_S yield > COS yield > CS_2_ yield^30^.

### Re-fixation of COS

**Figure 7:**
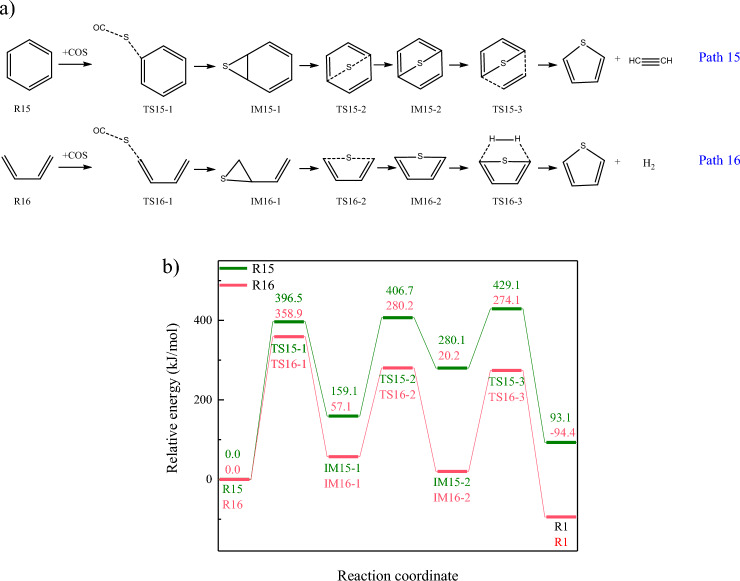
The combination of COS and organics. (**a**) Reaction Pathways (**b**) Changes of relative energies in path15-16.

During CO_2_ gasification of carbon-based fuel, thiophene will be decomposed into gaseous sulfur, meanwhile, gaseous sulfur will also be captured by nascent char to form thiophene in char^31^, while thiophene is the dominate solid sulfur in char^32^, indicating the re-fixation of the dominate gaseous sulfur (COS) will be converted into thiophene. The introduction of CO_2_ leads to the generation of carboxyl functional groups in char, and H_2_S will be captured by carboxyl functional groups through dehydration reaction^14^. Generated solid sulfur with poor thermal stability will further be converted into thiophene, through aromatization reaction. Moreover, gaseous sulfur will also be captured by unsaturated functional groups. Unsaturated bond (C=S bond) in COS is prone to combine with unsaturated bonds in char, forming quaternary-ring or hexagonal-ring organics. However, thiophene is the organic sulfur with five-membered ring structure, indicating the cyclic products generated from addition reactions, will be converted into thiophene through ring-opening and re-cyclization reactions that with high energy barrier. Therefore, the addition of COS and organics in char tends to form sulfur-containing heterocycles, instead of thiophene. Besides, sulfur in COS will be transferred to organics in char, through de-carbonylation reaction, as shown in Fig. 7a. Furthermore, sulfur will be transferred from adjacent carbon to para carbon, and thioether will be converted into five-membered ring, which will be converted into thiophene ring through further polycondensation.

According to Fig. 7b, sulfur migration between COS and organics are the rate-determining steps in both path15-16. The energy barriers for capturing COS by aliphatics and aromatics are 358.9 kJ/mol and 396.5 kJ/mol, respectively. Energy barriers for converting COS to thiophene are lower than those of thiophene decomposition, indicating the conversion of COS to thiophene is an effective way for in-situ sulfur fixation. Furthermore, Gibbs free energy change for path16 is negative, while that of path15 is positive, indicating COS tends to be attached to aliphatic chains and converted to thiophene.

Therefore, during CO_2_ gasification, the re-fixation of COS will not occur on aromatics in char directly. Aromatic ring will be converted into aliphatic chains, through the reaction with CO_2_. COS will be captured by unsaturated aliphatic chains. As the proceed of CO_2_ gasification, the aromaticity of organics in char increases, promoting the conversion of solid sulfur to thiophene and inhibiting the release of sulfur, but meanwhile weakening the sulfur-fixation ability of organics in char.

## Conclusion

DFT (density functional theory) calculations of conversion between thiophene and COS during CO_2_ pyrolysis were analyzed in ORCA program with function M06-2X and basis set def2-SVP/def2-TZVP. The molecular properties analysis was also performed to determine the active sites on reactants. The conclusion can be summarized as follows:

1. The attachment of CO_2_ will lead to the destruction of the thiophene ring and the generation of COS, and CO_2_ adsorption is the rate-determined step. CO_2_ tends to be attached to the carbon atom that adjacent sulfur atoms, forming COS through the CO removal and hydrogen transfer. Though the non-aromatic structures formed by thiophene pyrolysis are more conducive for CO_2_ adsorption, the high-energy intermediates increase the overall energy barrier of the reaction pathway, and thus CO_2_ tends to be adsorbed directly onto the thiophene ring.
2. CO_2_ will be combined with thiophene ring through addition reaction. The combination of CO_2_ and thiophene will lead to the generation of COS and CO, which is thermodynamically feasible at 1173K. CO_2_ tends to be combined with thiophene through addition reaction, rather than CO_2_ adsorption reaction, and the energy barrier of reactions occurring on the aliphatics are lower than that occurring on the aromatics.
3. Gaseous sulfur will be converted to COS during CO_2_ gasification. CS_2_ will be converted into H_2_S and COS, with the combination with CO_2_/H_2_O. But H_2_S will not be converted into COS at 1173K, and thus the yield of H_2_S is higher than that of COS during CO_2_ gasification.
4. COS will be captured by char, forming solid organic sulfur. The re-fixation of COS will not occur on aromatics in char directly. Aromatic ring will be converted into aliphatic chains, through the reaction with CO_2_. COS will be captured by unsaturated aliphatic chains. As the proceed of CO_2_ gasification, the aromaticity of organics in char increases, promoting the conversion of solid sulfur to thiophene and inhibiting the release of sulfur, but meanwhile weakening the sulfur-fixation ability of organics in char.

## Data Availability

All data generated or analysed during this study are included in this published article.
